# Idiopathic giant adrenal calcification: a rare case report

**DOI:** 10.3389/fonc.2024.1375748

**Published:** 2024-07-03

**Authors:** Zhiqiang Ji, Dalong Song, Hua Zuo, Xiaolong Chen, Wei Ji, Jiajun Yang, Qing Wang, Kehua Jiang

**Affiliations:** ^1^ Department of Urology, Guizhou Provincial People’s Hospital, Guiyang, Guizhou, China; ^2^ Department of Graduate School, Zunyi Medical University, Zunyi, Guizhou, China; ^3^ Department of Urology, The First People’s Hospital of Qingzhen, Guiyang, Guizhou, China; ^4^ Department of Pulmonary and Critical Care Medicine, The Second Clinical College of Fujian Medical University, Quanzhou, Fujian, China; ^5^ Department of Graduate School, Guizhou Medical University, Guiyang, Guizhou, China

**Keywords:** adrenal gland, case report, epigastric pain, giant adrenal calcification, retroperitoneoscopic

## Abstract

**Background:**

We describe a rare case of giant adrenal calcification as the main cause of sudden onset epigastric pain in a 57-year-old female patient.

**Case description:**

Computed tomography (CT) of the whole abdomen in this patient showed calcified foci measuring approximately 7.8 × 5.4 × 7.1 cm in the hepatorenal recess, and no enhancement effect was seen. Secondary causes of adrenal calcification in this patient were ruled out, and a rare diagnosis of a primary giant adrenal calcification was made. Subsequently, the right adrenal gland and calcified mass were completely resected. The calcification did not recur during 6 months of follow up.

**Conclusions:**

Although other cases of adrenal calcification of unknown origin have been reported, cases of giant idiopathic adrenal calcification are rare. In this case, huge calcification of the right adrenal gland caused abdominal pain, which disappeared after the mass was excised. The etiology, pathogenesis, clinical symptoms, and prognosis of idiopathic adrenal calcification are still unclear. Additional case reports are needed to gain a better understanding of the diagnosis and treatment of this condition.

## 1 Introduction

Adrenal calcification can be secondary to a variety of adrenal diseases in addition to the more common chronic granulomatous infection caused by tuberculosis, including pheochromocytoma and adrenal hemorrhage, which can be secondary to adrenal myelolipoma and adrenal cysts. These causes are common in adults. In contrast, adrenal calcification due to adrenocortical carcinoma, neuroblastoma, and Wolman’s disease, are commonly observed in children or infants. Rare diseases such as calcifying fibrous tumor of the adrenal gland, adrenal hemangiomas, and adrenal mature teratoma can also cause adrenal calcification (1).

With the development of diagnostic imaging, an increasing number of incidentally detected adrenal calcifications are being detected (2). However, idiopathic giant adrenal calcification is very rare clinically. This case report describes a case of idiopathic giant adrenal calcification.

## 2 Case description

**Figure 1:**
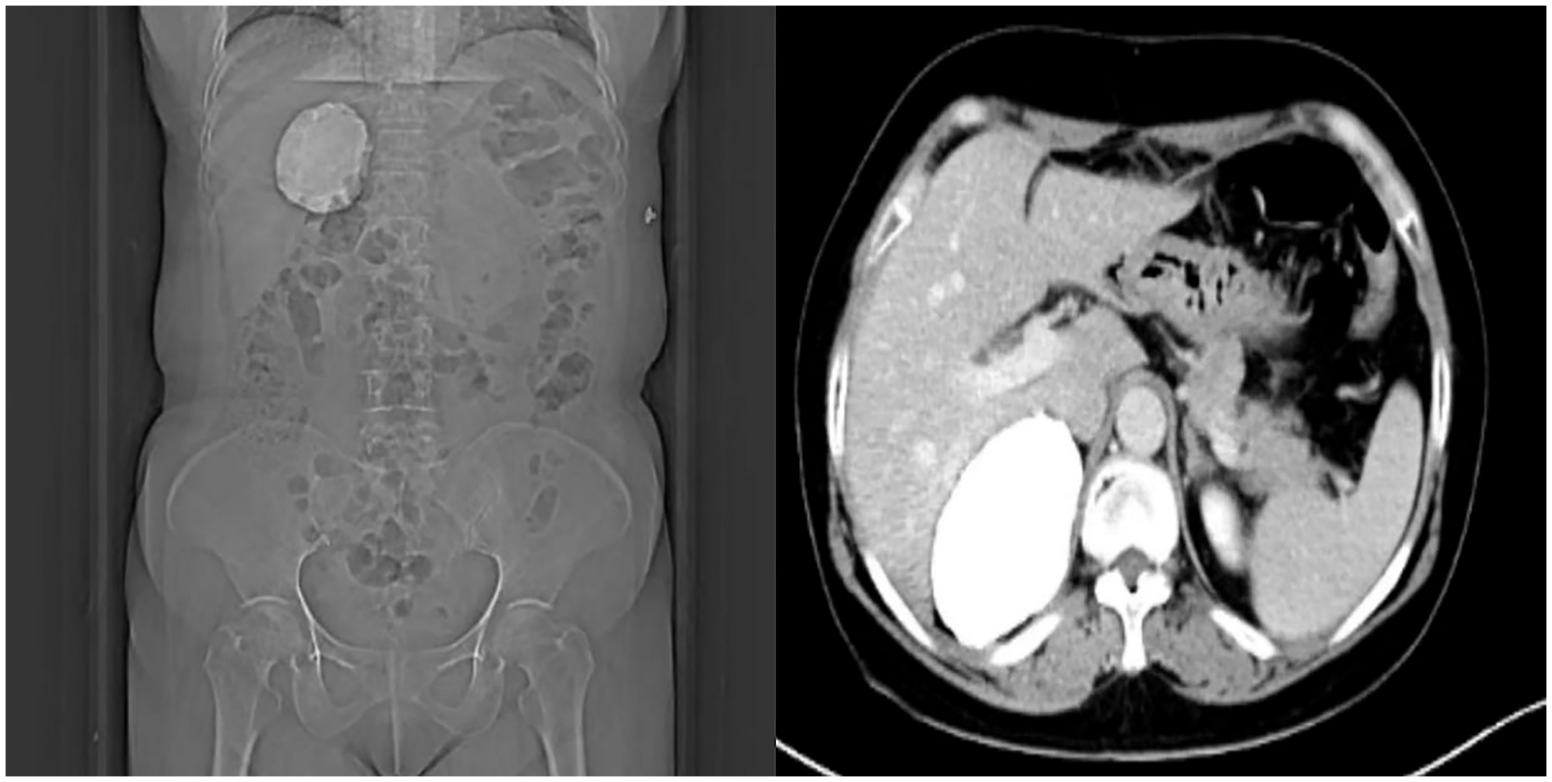
Upper abdominal computed tomography (CT) showing a hyperdense shadow in the hepatorenal recess, with a CT value of 700–1400 HU, and size of approximately 78×54×71 mm, without enhancement on enhancement scanning.

A 57-year-old female patient with sudden onset of epigastric pain lasting three days after eating spicy and stimulating food presented to the emergency department of our hospital. Computed tomography (CT) of the whole abdomen (**Figure 1**) revealed a 7.8 × 5.4 × 7.1 cm large calcified lesion with clear borders in the hepatorenal recess No enhancement of the lesion was seen on the enhancement scan. Laboratory and endocrine tests did not reveal any apparent abnormalities.

Physical examination suggested that the patient had significant subxiphoid pressure pain but no percussion pain in either renal region. Moreover, she had no history of tuberculosis, anticoagulant medication ingestion, trauma, surgery, or family history of hereditary disease. Other causes of adrenal calcification were ruled out after a multidisciplinary consultation, including granulomatous disease, adrenal cysts, adrenal calcifying fibroadenomas, neuroblastomas, pheochromocytomas, adrenocortical carcinomas, adrenal hemorrhages, and adrenal myelolipomas.

**Figure 2:**
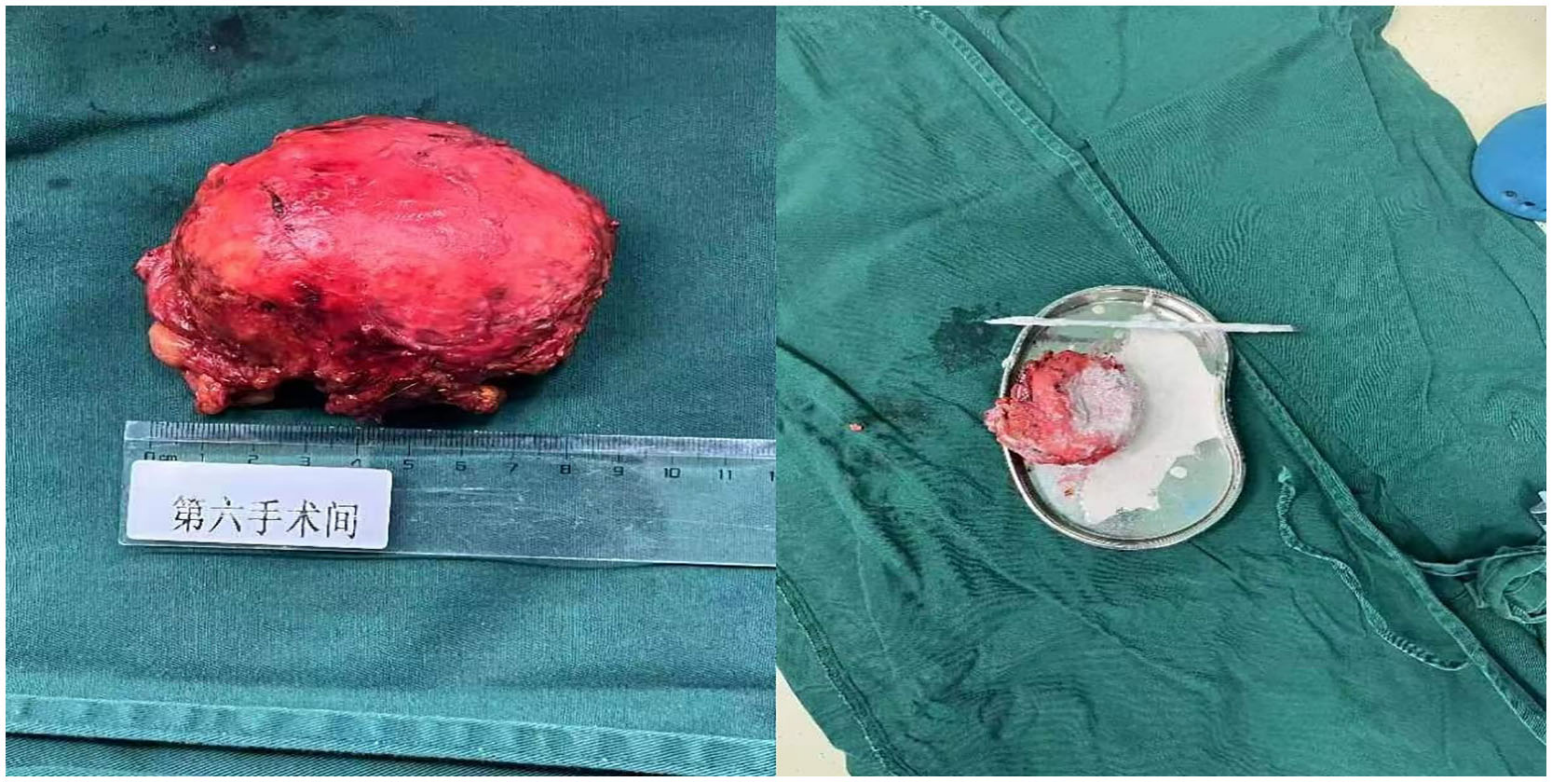
The pathological specimen broadly suggests that the right adrenal gland occupying tissue is a piece of grayish-white elliptical tissue, with a smooth surface, intact peripheral membrane, and the cystic capsule exhibits a wall thickness of 0.1–0.2 cm in section, feels palpably hard, and the capsule cavity is filled with a white gypsum-like material.

**Figure 3:**
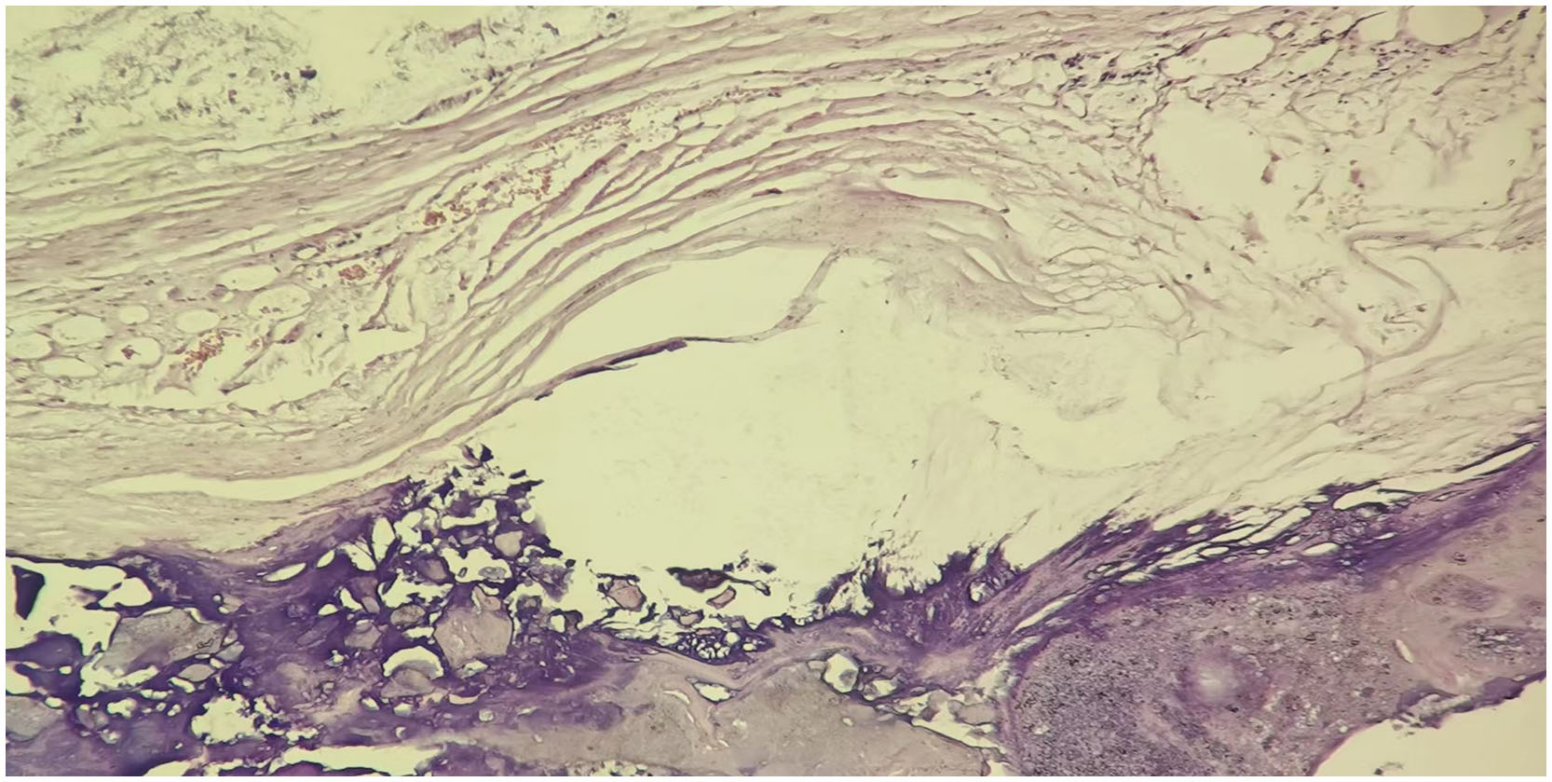
Hematoxylin and eosin staining of the pathological specimen reveals that the neoplasm of the right adrenal gland shows cystic changes, the wall of the capsule is calcified tissue, and the capsule is filled with patches of necrotic tissue.

**Figure 4:**
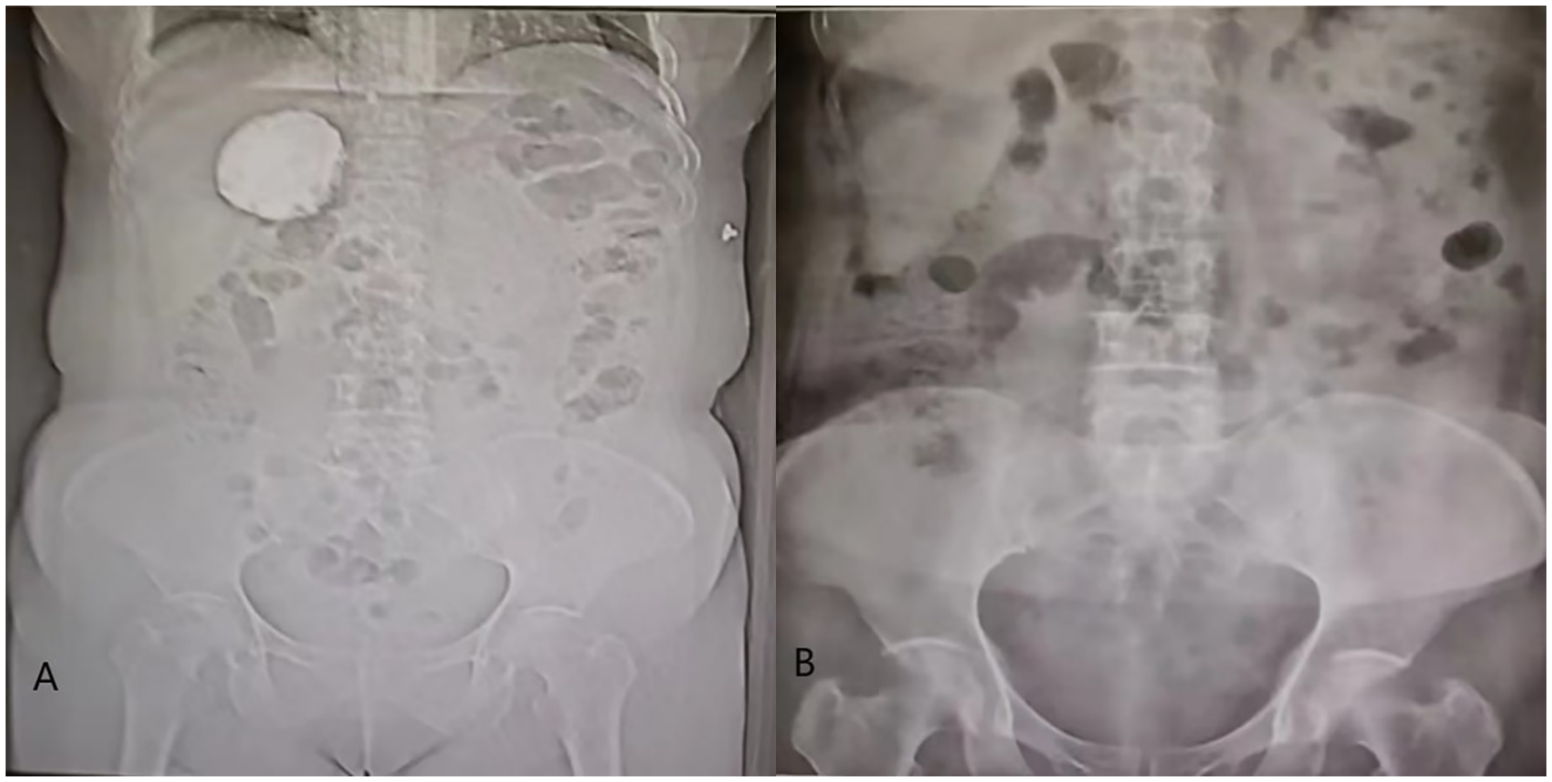
**(A)** shows an obvious calcification in the liver-kidney recess before operation, and figure **(B)** shows no obvious calcification in the hepatorenal recess after operation.

Given that the lesion was > 4 cm in diameter, the patient’s symptoms of abdominal pain, and the diagnosis of idiopathic giant adrenal calcification in the right adrenal gland, we decided to treat this patient with surgical resection. Because conventional open surgery is highly invasive and malignancy had been ruled out, we decided to perform a laparoscopic adrenalectomy (3, 4), after discussing the options with the patient and her family. We chose posterior laparoscopic access (5) according to the surgeon’s habits. We used the lateral approach via the posterior peritoneum, with the patient in the left lateral position. The extraperitoneal fat was separated by ultrasonic incision, and the perirenal fascia and perirenal fat were incised. The right perirenal tissue was then separated and the right kidney was freed. Exploration of the upper pole of the right kidney revealed a large mass that was tightly adherent to the adrenal gland. The mass was hard with a stoney consistency, clearly defined, with no obvious adhesion to the liver, peritoneum, or upper pole of the kidney. The tissues surrounding the mass were separated and the mass was freed. The central vein of the right adrenal gland was then separated and ligated with vascular ligature clips and then dissected, and the right adrenal gland and mass were completely resected. The operative time was 155 minutes and blood loss was100 mL. Gross inspection of the resected specimen revealed a space-occupying mass of the right adrenal gland measuring 8 × 6.5 × 4 cm. The mass was firm in texture, with a milky-white effusion, some caseous exudate visible in the incision, and foci of milky-white calcification visible in the section (**Figure 2**). Histopathology revealed cystic changes of the cut surface of the mass, with calcified tissue in the wall of the capsule. Large deposits of calcium salts were seen in the lumen of the capsule without tissue structure, consistent with adrenal calcification (**Figure 3**). The postoperative diagnosis was idiopathic giant adrenal calcification. We positioned a drainage tube in the subhepatic space and left it in place for 72 hours. Postoperatively, the patient’s condition stabilized, her epigastric pain decreased significantly, and she was discharged from the hospital on day 7 postoperatively. Abdominal radiography confirmed that the mass had been completely removed (**Figure 4**). Follow-up to date has not revealed a recurrence of the calcification or any other abnormalities. Follow up of the patient is continuing.

## 3 Discussion

Calcification is the focus of cellular necrosis that occurs in body tissues in response to metabolic or infectious factors, with calcium deposited at the site of cellular damage and necrosis. Most clinical adrenal calcifications are discovered incidentally during CT for other purposes. In a retrospective single-center study that reported on the etiology of adrenal calcification in 540 adult patients, approximately 20% of the adrenal calcifications were secondary to tumors, 5% were due to adrenal hemorrhages, and 1% were caused by infectious factors such as tuberculosis; in the remaining cases, the etiology of adrenal calcification was unknown and was classified as idiopathic calcification (6). Idiopathic giant adrenal calcifications are clinically rare and have been rarely reported worldwide (7, 8). Patients with this type of adrenal calcification had no previous birth injuries, trauma, or other predisposing factors for adrenal hemorrhage. They are also mostly asymptomatic, and when symptoms occur, they are mostly related to the size and location of the lesions, including abdominal pain or the presence of a palpable mass. Laboratory and endocrine tests were normal in these patients, and imaging studies often showed dense calcified shadows on CT, with no associated concomitant adrenal disease (9). The European Society of Endocrinology Clinical Practice Guidelines, in collaboration with the European Network for the Study of Adrenal Tumors for idiopathic adrenal calcification can be consulted for the principles of treatment (10).

In this case, the levels of circulating hormones such as plasma cortisol and urinary catecholamines were normal. In addition, the patient had no history of sarcoidosis elsewhere in the body and repeated tuberculin tests were negative. Therefore, we excluded granulomatosis, neuroblastoma, pheochromocytoma, and other functional adenomas, which are most commonly associated with tuberculosis. The lack of significant enhancement and vascular invasion on imaging provided no evidence of a nonfunctioning adrenal gland, adrenal malignancy, or hemangioma. Additionally, this patient had no history of coagulopathy or trauma, to suggest that calcification could have not considered secondary to adrenal bleeding. Based on consideration of the patient’s medical history, family history, imaging, and laboratory test results, she was diagnosed with idiopathic giant adrenal calcification after a multidisciplinary consultation.

This patient was treated with surgical resection, considering that the lesion was >4 cm large and the patient was experiencing symptoms of abdominal pain. The patient’s epigastric pain symptoms significantly improved after the procedure. Calcification of the adrenal gland has been reported in most adrenal diseases, including tuberculosis, hematomas, cysts, and benign and malignant tumors (11). However, large idiopathic adrenal calcifications, as observed in our patient, have rarely been reported. We suspect that this primary adrenal calcification may be related to two factors. First, we believe that it may be related to the physiological endocrine hormone disruption caused by the patient’s poor lifestyle, mental stress, and stress reactions (12). Second, the patient had no obvious daily symptoms such as anemia or pain, but we could not exclude the possibility that the patient had adrenal vascular abnormalities such as hemangiomas prior to disease onset. Based on cautious speculation, we believe that spontaneous adrenal hemorrhage followed by calcification cannot be excluded for this patient (13). However, even if calcification is induced, giant adrenal calcifications are rare. We would like to further trace the patients’ family associated adrenal vascular diseases. Unfortunately, the patient and her family refused further testing, which was the main limitation of this study. Additional case studies will be needed in the future to help explore the risk factors and mechanisms underlying the pathogenesis of idiopathic giant adrenal calcifications in patients with no prior history of tuberculosis, trauma, other relevant medical conditions, or adrenal dysfunction with tumorigenesis. In this case, the size of the mass led to it manifesting as abdominal pain, which may have been caused by the mass pressing on nerves or causing peritoneal irritation. Because little is known about the natural history of giant adrenal calcifications, lifelong follow-up is recommended.

## Data availability statement

The original contributions presented in the study are included in the article/supplementary material. Further inquiries can be directed to the corresponding authors.

## Ethics statement

Written informed consent was obtained from the individual(s) for the publication of any potentially identifiable images or data included in this article.

## Author contributions

ZJ: Investigation, Data curation, Writing – review & editing, Writing – original draft. DS: Funding acquisition, Writing – review & editing. HZ: Writing – original draft, Data curation. XC: Writing – original draft. WJ: Writing – review & editing, Writing – original draft. JY: Writing – original draft. QW: Writing – review & editing, Writing – original draft. KJ: Funding acquisition, Writing – review & editing, Writing – original draft.
